# Pharmacokinetics of Intravenous and Transdermal Flunixin Meglumine in Wool and Hair Sheep (*Ovis aries*)

**DOI:** 10.1111/jvp.70015

**Published:** 2025-08-01

**Authors:** Kaitlyn G. Forrest, Jennifer L. Halleran, Ronald E. Baynes, Danielle A. Mzyk

**Affiliations:** ^1^ Department of Population Health and Pathobiology North Carolina State University College of Veterinary Medicine Raleigh North Carolina USA

**Keywords:** analgesia, flunixin, pharmacokinetics, sheep, transdermal

## Abstract

The objective of the study was to evaluate the pharmacokinetics of flunixin meglumine of intravenous (IV) and transdermal (TD) flunixin meglumine administration on different coat types (wool vs. hair) in 12 healthy sheep. Polled dorset (wool) sheep (*n* = 6) and katahdin (hair) sheep (*n* = 6) received 2.2 mg/kg IV and 3.3 mg/kg TD with a 10‐day washout period between treatments. Plasma samples were obtained for 96 h following both IV and TD administration, respectively. Flunixin concentrations were quantified by use of high‐performance liquid chromatography with mass spectrometry, and PK parameters were derived using different modeling techniques. A population non‐linear mixed effect model showed that coat type has a significant effect on the absorption rate following TD administration. The mean bioavailability of TD flunixin was not significantly different (48.76% ± 17.49% and 36.61% ± 4.33%; *p* = 0.093) in wool and hair sheep, respectively. Maximum plasma concentrations following TD administration were higher in wool sheep (1.57 μg/mL; range, 0.6–3.41 μg/mL) compared to hair sheep (0.57 μg/mL; range, 0.36–0.83 μg/mL). The PK results provide further support for clinical studies to examine the efficacy of TD flunixin in different breeds of sheep.

## 1 Introduction

Flunixin meglumine is a non‐steroidal anti‐inflammatory drug (NSAID) that is commonly used in veterinary medicine for its anti‐inflammatory, analgesic, and antipyretic properties. Like other NSAIDs, flunixin reduces inflammation by inhibiting cyclooxygenase and, in turn, decreasing the production of inflammatory mediators (Cheng et al. 1998). Flunixin has been shown to be an effective analgesic in sheep (Welsh and Nolan 1995), there are currently no drugs approved for the control of pain in sheep in the United States, and very limited NSAIDs approved for use in food‐producing species. A transdermal (TD) formulation of flunixin meglumine has been approved for cattle for control of pain associated with footrot, demonstrating rapid absorption and therapeutic effectiveness (Food and Drug Administration 2017). There is a need to explore the potential of this transdermal formulation in different sheep breeds, which may provide veterinarians and producers a more practical method of providing pain relief when compared to intravenous injection.

A major concern for all pharmacokinetic experiments is intra‐ and inter‐subject variability. In veterinary medicine, the characterization of a drug's pharmacokinetic (PK) properties is generally based upon data that are derived from studies that use small groups of healthy animals, often of a single breed (Martinez and Modric 2010). The goal of therapeutic dosing is to achieve the highest possible systemic bioavailability and limit variability (Toutain and Bousquet‐Mélou 2004). Bioavailability is defined as the extent and the rate at which a substance or its active drug is delivered from a pharmaceutical form and becomes available in systemic circulation (Toutain and Bousquet‐Mélou 2004). The transdermal route of administration offers a more easily accessible dosing strategy for producers and veterinarians; however, species differences in absorption, distribution, metabolism, and elimination must be examined. Goats and alpacas administered a single dose of TD flunixin meglumine demonstrated a low bioavailability at 24.76% and 25.05%, respectively, when compared to 48% in calves (Reppert, Kleinhenz, Montgomery, Bornheim, et al. 2019; Kleinhenz et al. 2018; Reppert, Kleinhenz, Montgomery, Heiman, et al. 2019). In addition, both goats and alpacas showed slow absorption, poor distribution, and prolonged elimination half‐life as compared to cattle (Reppert, Kleinhenz, Montgomery, Heiman, et al. 2019; Reppert, Kleinhenz, Montgomery, Bornheim, et al. 2019; Kleinhenz et al. 2018; Meira et al. 2022). To date, there have been no studies evaluating the pharmacokinetics of transdermal flunixin meglumine or its bioavailability in sheep. Due to the variability of breeds of sheep across the United States, there is a critical need to evaluate transdermal flunixin meglumine across different coat types (wool vs. hair) when using a transdermal formulation. Therefore, the objective of this study was to determine the pharmacokinetic properties and bioavailability of TD flunixin meglumine in healthy adult ewes of different breeds/coat types (polled dorset and katahdin). The authors hypothesize that hair sheep (katahdins) will have higher systemic bioavailability when compared with wool sheep (dorsets) but similar PK parameters when compared to goats.

## 2 Materials and Methods

### 2.1 Animals

Twelve (six polled dorset [wool breed]; six katahdin [hair sheep]) ewes sourced from the North Carolina State University Small Ruminant Education Unit. For the enrolled polled dorset ewes, ages ranged between 8 months and 4 years (mean ± standard deviation; 1.2 ± 1.2 years) with an average weight of 50 ± 13.6 kg. For the katahdin ewes, ages ranged between 8 months and 1 years (mean ± standard deviation; 0.8 ± 0.1 years) with an average weight of 42 ± 4.1 kg. Inclusion criteria included non‐pregnant adult ewe sheep, healthy via physical examination by the corresponding author (D.A.M.) and lab animal veterinarian, and no recent drug administration prior to enrollment. Wool sheep were not sheared prior to enrollment or during the study. All ewes were group housed inside the Laboratory Animal Resources facility at North Carolina State University in a temperature‐controlled room (temperature of the room where the sheep were housed averaged 71°F across study period). The diet for all sheep included ad libitum grass hay and water. The study protocol was approved by the Institutional Animal Care and Use Committee (IACUC) of North Carolina State University (Protocol #23‐315‐01).

### 2.2 Study Design

Polled dorset (wool) sheep (*n* = 6) and katahdin (hair) sheep (*n* = 6) either received 2.2 mg/kg flunixin meglumine IV (50 mg/mL, Hospira Inc. Lake Forest, IL USA) and 3.3 mg/kg TD (Banamine Transdermal, Merck Animal Health) with a 10‐day washout period between treatments. Twenty‐four hours prior to the study, a sterile 16‐gauge jugular catheter was placed to facilitate serial blood sampling in a jugular vein. For the intravenous study, flunixin was administered through a temporary IV catheter in the opposite jugular vein from the sampling catheter. For TD administration, a single dose of flunixin was applied along the dorsal midline from the cervicothoracic junction to the mid‐lumbar region using a disposable single‐use syringe. The wool/hair was parted by hand to ensure adequate skin contact with TD flunixin. A separate individual who did not collect any samples dosed the sheep to prevent cross‐contamination. Waste blood (5 mL, representing 3× the catheter volume plus extension set residual volume) was collected from the jugular catheter into a syringe and discarded. Blood samples (6 mL) were then collected from the jugular catheter and immediately placed into plastic vacutainer tubes containing lithium heparin (BD Vacutainer, Franklin Lakes, NJ). Baseline samples were collected at 0 (predrug administration). For IV administration, samples were collected more frequently earlier in the study (5, 10, 15, 20, 30, 45, and 60 min) as compared to TD (15, 30, 45, and 60 min). Both routes of administration were sampled at 1.5, 2, 4, 6, 8, 10, 12, 24, 36, 48, 60, 72, 84, and 96 h after administration. Within 1 h of collection, all blood samples were centrifuged at 3000×*g* for 10 min. Each plasma sample was collected, transferred into 2 mL cryogenic plastic storage tubes, and stored at −80°C until sample analysis.

### 2.3 Plasma Flunixin Analytical Methods

#### 2.3.1 Chemicals and Reagents

The reagents were of LC/MS grade. Methanol (MeOH), acetonitrile (ACN), and formicacid were supplied by Fisher Chemical (Raleigh, NC, USA) and phosphoric acid was supplied by Aldrich Chemistry (Burlington, MA, USA). The flunixin meglumine reference standard was purchased from Sigma‐Aldrich (St. Louis, MO, USA). The ultrapure water was supplied by Synergy water purification system (Millipore). Analytical analysis of flunixin was carried out via ultra‐performance liquid chromatography (UPLC) and tandem mass spectrometric (MS/MS) detection (Waters Corporation, Milford, MA, USA). The UPLC‐MS/MS system consisted of an Acquity UPLC I class Binary Solvent Manager, Acquity UPLC Sample Manager FTN, and a Xevo TQD tandem mass spectrometer (Waters Corporation, Milford, MA, USA).

#### 2.3.2 UPLC‐MS/MS Conditions

Chromatographic separation was performed by a gradient elution on the ACQUITY UPLC BEH phenyl 1.7 μm column (2.1 × 100 mm) with a VanGuard pre‐column (Waters Corporation, Milford, MA, USA). The mobile phase solvents were 0.1% formic acid in water (A) and 0.1% formic acid in ACN (B) at a flow rate of 0.4 mL/min for 5 min. The gradient program mobile phase conditions were 70% of A and 30% of B for the first 2.5 min, then changed linearly to 10% of A and 90% of B from 2.5 to 3.5 min, then immediately back to 70% of A and 30% of B from 3.5 to 5 min to re‐equilibrate at the initial conditions. The column temperature was 35°C and the autosampler temperature was maintained at 25°C. Two standard curves were constructed ranging from 0.0005 to 1 μg/mL (lower standard curve) and from 0.05 to 10 μg/mL (higher standard curve). The injection volume was 0.5 μL for the lower plasma standard curve (0.0005–1 μg/mL). The injection volume was 0.1 μL for higher concentration plasma standard curves (0.05–10 μg/mL). Positive electrospray ionization (ESI [+]) was used with multiple reactions monitoring (MRM). The true page source voltages were 3.2 kV and 40 V for the capillary and cone, respectively. The source desolvation temperature was 500°C. The source desolvation gas flow was 1000 L/h and the cone gas was 50 L/h. The MS file cone voltage setting was 44 V with a collision energy setting of 34 and 22 V. Argon was used as the collision gas and nitrogen was used as the desolvation and cone gases. Quantification was performed using the transition Parent (m/z): 297.10 and Daughter (m/z): 263.99 and 279.09 with a retention time of 2.44 min.

### 2.4 Sample Analysis

#### 2.4.1 Plasma

One hundred microliter of plasma was pipetted into the 2.0 mL microcentrifuge tubes low retention (Thermo scientific, CA, USA). 500 μL of 4% phosphoric acid prepared in water was added to each tube to pretreat the plasma. Solid phase extraction was performed on an oasis prime HLB 96 well μ Elution plate (Water Corporation, Milford, MA, USA) with a positive pressure manifold, Otto specialist (Waters Corporation, Milford, MA, USA). The plate was prepared by conditioning the plate with 500 μL of MeOH followed by 500 μL of ultrapure water. After that, 600 μL of pretreated plasma was loaded on the prime HLB μElution plate and passed through the plate by using the positive pressure manifold, Otto specialist (Waters Corporation, Milford, MA, USA). Then, the plate was washed with 600 μL of 5% MeOH prepared in water. The analyte was eluted into a clean 96 well sample plate (700 μL round 96 well samples plate, Water Corporation, Milford, MA, USA) with the addition of 100 μL of an elution solution (70:30:ACN:MeOH) and eluents were injected into the instrument for analysis. Standards from 0.0005 to 1 μg/mL (lower standard curve) and from 0.05 to 10 μg/mL (higher standard curve) were prepared using blank sheep plasma. The appropriate standard curve was selected to quantify the flunixin concentrations in samples. For the higher standard curve (0.05–10 μg/mL of flunixin) and samples that fit within this range, 400 μL of ultra‐pure water was added to 100 μL eluents and mixed thoroughly prior to analysis. The 0.083‐, 0.16‐, 0.25‐, 0.33‐, and 0.5‐h samples from the IV group were diluted two times with blank sheep plasma prior to sample preparation because concentrations exceeded the upper limit (10 μg/mL) of the higher standard curve. The calibration curve of flunixin was fitted with a weighted (1/concentration) linear equation by using Targetlynx software. The calibration ranges of 0.0005–1 and 0.05–10 μg/mL were linear with a coefficient of determination, *R* ^2^, greater than 0.99. Each calibration standard concentration could be back calculated to within 15% of the true concentration. To validate the analytical method, a total of five replicates at concentrations of flunixin (0.001, 0.003, 0.03, and 0.7 μg/mL for calibration curve of 0.0005–1 μg/mL) and (0.1, 0.3, 3, and 7 μg/mL for calibration curve of 0.05–10 μg/mL) were tested. The intra‐day precision and accuracy were calculated. The precision was 4.1%–7.6%, and recovery was 90.1%–107.8% (Table S1). The limit of detection and limit of quantification was recognized as 0.0001 and 0.001 μg/mL for standard curve (0.0005–1 μg/mL) and 0.05 and 0.1 μg/mL for standard curve (0.05–10 μg/mL) based on precision and accuracy, signal to noise ratio, and chromatograph, respectively. Inter‐day precision and accuracy are shown in Table S2.

### 2.5 Pharmacokinetic Analysis

Phoenix WinNonlin version 8.3 (Certara Inc., Princeton, NJ, USA) was used to determine pharmacokinetic parameters from plasma flunixin concentration‐time data for each individual sheep using noncompartmental methods based on statistical moment theory as well as compartmental analysis. Individual animal pharmacokinetics were determined for each route of administration, and descriptive statistics are reported. The peak plasma concentration (*C*_max_) and time to obtain peak concentration (*T*_max_) were reported as observed values for the TD route, and initial concentration at time zero (*C*_0_) was determined for IV administration. From non‐compartmental analysis values, the rate constants associated with the terminal elimination phase (*λ*_*z*_) following TD and IV administration were estimated by using linear regression of the terminal phase of the log plasma concentration‐time curve, and the terminal elimination half‐lives (*t*_1/2_ *z*) were determined. The area under the plasma concentration‐time curve (AUC_0–∞_) and the area under the first moment of the concentration–time curve (AUMC_0–∞_) were calculated by extrapolating to infinity using the linear/logarithmic trapezoidal method. Absolute clearance (CL), apparent volume of distribution (*V*_z_), volume of distribution at steady‐state (*V*_ss_), and mean residence time (MRT_0–∞_) were calculated from AUC_0–∞_ and AUMC_0–∞_. Mean residence time (MRT_0–∞_)was calculated from the equation: AUMC_0–∞_/AUC_0–∞_. Mean absorption time (MAT) represents the difference between mean residence time following the transdermal route (MRT_TD_) and mean residence time of intravenous administration (MRT_IV_) and was calculated for individual sheep using the following equation:

$$\mathrm{MAT}={\mathrm{MRT}}_{\mathrm{TD}}-{\mathrm{MRT}}_{\mathrm{IV}}$$

Dose adjusted bioavailability (*F*%) following TD route of administration was calculated for each sheep using the equation (Toutain and Bousquet‐Mélou 2004):

$$F=\left(\frac{{\mathrm{AUC}}_{\mathrm{TD}}}{{\mathrm{AUC}}_{\mathrm{IV}}}\right)\times \left(\frac{{\text{Dose}}_{\mathrm{IV}}}{{\text{Dose}}_{\mathrm{TD}}}\right)\times 100$$

To evaluate differences in absorption and distribution rates, an open 1‐ (*n* = 1) or 2‐ compartment (*n* = 11) model was fit to each sheep based on the best fit on the basis of visual analysis for goodness of fit and by visual inspection of residual plots. From compartmental analysis, the absorption rate constant (*K*_01_) describes the rate of drug absorption into the central compartment (highly perfused tissues including liver and kidneys). The absorption half‐life (*t*_1/2abs_) can be calculated using *K*_01_ with the following formula (Toutain and Bousquet‐Mélou 2004):

$$t_{1/2\mathrm{abs}}=\frac{0.693}{K_{01}}$$

The rate constants *K*_12_ and *K*_21_ represent the first‐order rate transfer constants for the movement of drug from compartment 1 to compartment 2 (*K*_12_) and from compartment 2 to compartment 1 (*K*_21_). The tissue or peripheral compartments are composed of groups of tissues with lower blood perfusion and different affinities for flunixin, which typically include fat and muscle.

In order to examine if coat type was the source of variability in this population of sheep, the mean parameters from the compartmental analysis were used to obtain initial estimates for a population pharmacokinetic analysis using nonlinear mixed‐effects modeling (NLME). This approach allows for the determination of typical population parameter values (fixed effects) and the estimation of between‐subject variability (random effects). By evaluating the data using an NLME model, the addition of covariates was explored to the base model to determine if coat type could explain the source of variability. Various models were tested with different error structures to determine the best fit base model. Final model selection was based on goodness‐of‐fit plots, diagnostic plots of residuals, scatter plots of predicted versus observed values, and statistical significance between models using the minimum value of the objective function (MOF). The addition of breed as a categorical covariate showed a reduction in the minimum value of the objective function. The MOF is proportional to minus twice the log‐likelihood (−2LL) of the data. Therefore, a likelihood ratio allowed for the comparison of our base model and covariate model. The relationship between the pharmacokinetic parameters of interest and breed was evaluated using the Chi‐squared test, comparing the difference in objective functions between the basic model (without the covariate) and the model that included breed. Breed of sheep was included in the final model since it produced a minimum decrease in the objective function of 3.64 units (*p* = 0.05, 1 degree of freedom) and if it decreased the interindividual variability of the corresponding pharmacokinetic parameter of interest.

### 2.6 Statistical Analysis

The estimated sample size was based on practical considerations as well as the justification and results from previous transdermal flunixin studies in goats and cattle (Reppert, Kleinhenz, Montgomery, Bornheim, et al. 2019; Kleinhenz et al. 2018). Pharmacokinetic values were reported as geometric means, medians, and ranges, except for *t*_1/2*z*_ and *t*_1/2abs_ which are reported as harmonic mean and harmonic standard deviation. The normality of the data distributions for all pharmacokinetic parameters was evaluated using the Shapiro–Wilk test. Comparisons between the breeds were performed with unpaired *t*‐tests for parametric parameters andWilcoxon rank sum tests for nonparametric parameters. Statistical analysis was performed using GraphPad Prism v9.0.2 (San Diego, CA, USA). *p*‐values ≤ 0.05 between both breeds were considered statistically significant.

## 3 Results

Prior to drug administration, sheep were in healthy body condition and exhibited no abnormalities in physical exams performed on the day of the experiments. All sheep remained bright, alert, and responsive, with normal appetite and thirst during all phases of the study, including blood collection. Approximately 62 h after TD administration, one sheep developed diarrhea. A fecal egg count was performed and 9325 strongyle‐type eggs were noted. This sheep was dewormed orally with a single dose of moxidectin (0.2 mg/kg), albendazole (7.5 mg/kg), and levamisole (8 mg/kg) at 72 h after the initial TD application.

**FIGURE 1:**
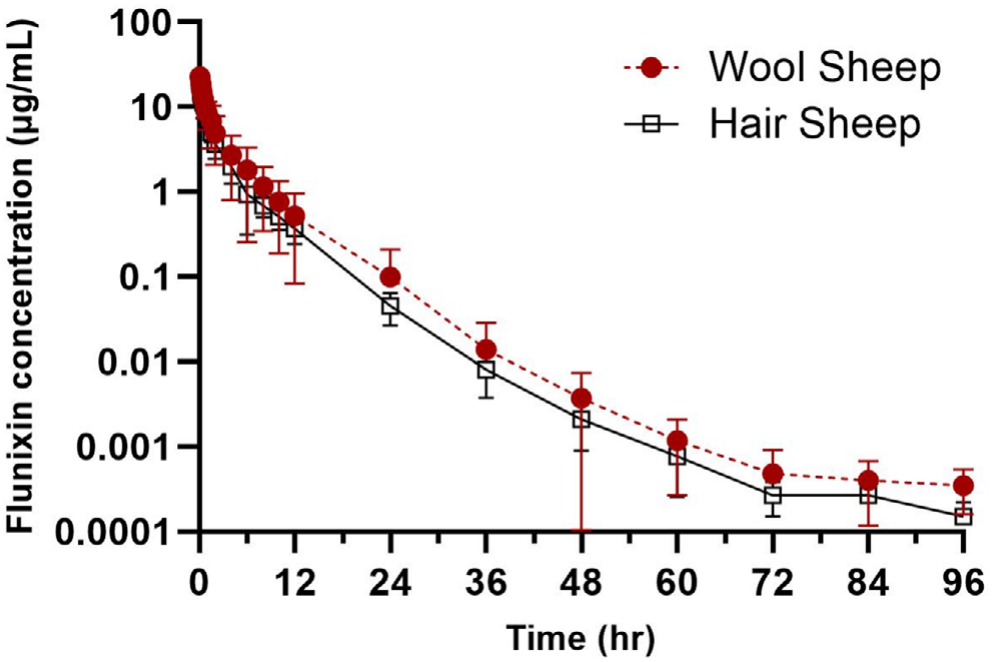
Plasma concentrations of flunixin over time (h) by intravenous (2.2 mg/kg) route in wool versus hair sheep.

**FIGURE 2:**
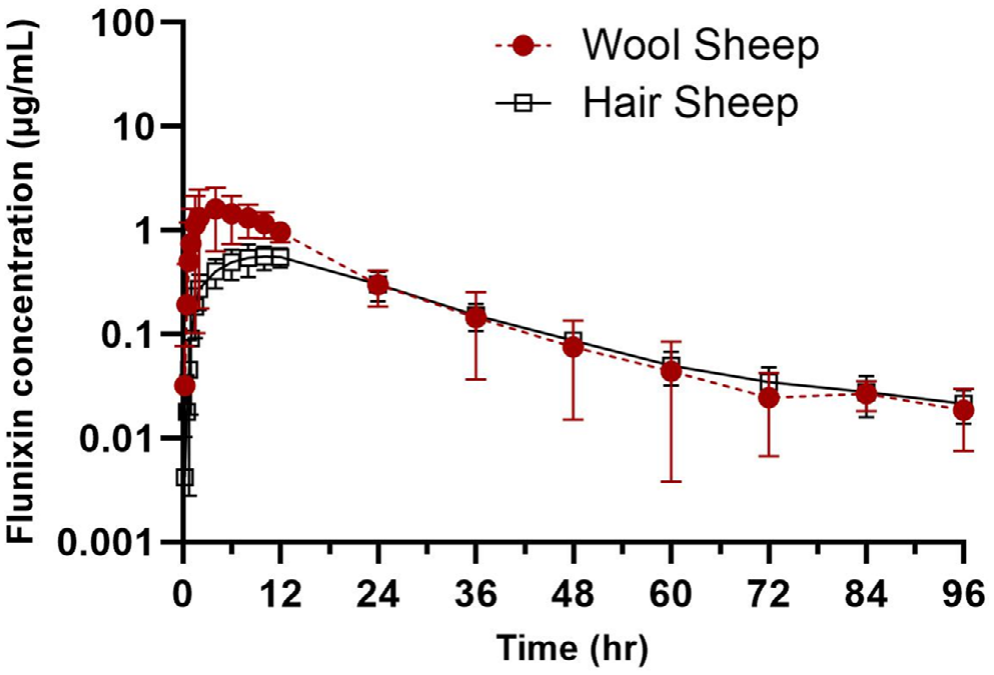
Plasma concentrations of flunixin over time (h) by transdermal (3.3 mg/kg) route in wool versus hair sheep.

The semi‐logarithmic plot of plasma flunixin concentrations over time after IV and TD administration for wool and hair sheep is presented in Figures 1 and 2. Pharmacokinetic parameters after IV dosing are presented in Table 1 and for TD in Table 2. The calculated bioavailability of TD administration was 48.8% and 36.31% for wool and hair sheep, respectively. Absorption half‐life and rate constants are presented in Table 3. The MAT was significantly (*p* = 0.045) decreased in wool sheep (13.74 h) compared to hair sheep (23.73 h). Using the NLME model, the typical values for the population (*θ*, theta) for absorption rate were 0.37 1/h and the coefficient of variation for flunixin in all sheep was 326.3%. When breed was entered as a covariate in the NLME analysis, there was a decrease in random source variability and an improved value of the MOF; therefore, it was included in the final model. Breed/coat type was found to have a significant effect on the variability and absorption rate following transdermal administration in sheep.

**TABLE 1:** Mean pharmacokinetic parameters of flunixin meglumine at the dosage of 2.2 mg/kg body weight following single intravenous (IV) administration in wool (dorset; *n* = 6) and hair (katahdin; *n* = 6) sheep using noncompartmental analysis.

Parameter	Unit	Wool (dorset) sheep				Hair (katahdin) sheep				*p*
		Mean	SD	Median	Range	Mean	SD	Median	Range	
*C*_0_	μg/mL	24.82	1.39	24.15	14.47–37.59	23.91	1.32	25.85	15.17–32.40	0.76
*z*	1/h	0.11	1.21	0.12	0.08–0.13	0.12	1.31	0.11	0.08–0.16	0.67
*t*_1/2_ *z* ^a^	h	6.36	1.13	6.01	5.32–9.16	5.99	1.54	6.25	4.40–8.55	0.79
AUC_0–∞_	h × μg/mL	41.84	1.69	41.35	22.45–85.54	30.69	1.13	31.48	26.20–34.69	0.16
AUC_extrapolated_	%	0.02	1.70	0.03	0.01–0.04	0.04	1.27	0.04	0.03–0.06	
AUMC_0–∞_	h^2^ × μg/mL	172.0	1.84	158.57	80.29–418.64	99.96	1.30	105.17	69.88–145.18	0.12
CL	mL/h/kg	52.58	1.59	53.89	25.72–98.01	71.70	1.13	69.98	63.43–83.98	0.22
*V*_ss_	mL/kg	216.1	1.42	232.54	125.86–350.59	233.6	1.26	223.79	187.75–345.94	0.77
*V*_*z*_	mL/kg	491.2	1.69	563.73	197.49–893.60	638.2	1.24	643.74	461.65–801.13	0.36
MRT_0–∞_	h	4.11	1.20	3.86	3.49–5.31	3.26	1.24	3.21	2.59–4.19	0.07

Abbreviations: AUC_0–∞_, area under the plasma concentration time curve from time 0 to infinity; AUMC_0–∞_, area under the moment curve from time 0 to infinity; Cl, total body clearance; *C*_0_, concentration at time zero; MRT_0–∞_, mean residence time; *t*_1/2*λz*_, elimination half‐life; *V*ss, volume of distribution at steady state; *V*_*z*_, apparent volume of distribution during terminal phase; *λ*_*z*_, elimination rate constant.

^a^ Harmonic mean and harmonic standard deviation.

**TABLE 2:** Mean pharmacokinetic parameters of flunixin meglumine at the dosage of 3.3 mg/kg body weight following single transdermal (TD) administration in wool (dorset; *n* = 6) and hair (katahdin; *n* = 6) sheep using noncompartmental analysis.

Parameter	Unit	Wool (dorset) sheep				Hair (katahdin) sheep				*p*
		Mean	SD	Median	Range	Mean	SD	Median	Range	
*z*	1/h	0.05	1.28	0.06	0.04–0.07	0.04	1.27	0.04	0.03–0.05	0.048
*t*_1/2_ *z* ^a^	h	12.57	0.22	11.93	10.07–18.76	17.43	0.22	17.33	13.15–27.36	0.063
*T*_max_	h	4.99	1.95	4.00	2.00–12.00	9.34	1.34	6.00	8.00–12.00	0.08
*C*_max_	μg/mL	1.57	1.77	1.63	0.6–3.41	0.57	1.33	0.59	0.36–0.83	0.0087
AUC_0–∞_	h × μg/mL	27.94	1.22	27.3	21.8–38.3	16.74	1.18	17.23	13.69–20.86	0.002
AUC_extrapolated_	%	1.05	2.69	0.98	0.25–4.34	3.08	1.82	3.10	1.29–7.90	
AUMC_0–∞_	h^2^ × μg/mL	465.0	1.41	392.8	332.1–740.5	442.8	1.34	483.0	248.03–539.2	0.818
MRT_0–∞_	h	16.64	1.53	14.3	10.2–31.9	26.44	1.27	26.4	17.74–37.4	0.06
*F*	%	44.51	1.67	53.22	16.97–73.37	36.37	1.13	35.56	32.70–45.99	0.093

Abbreviations: AUC_0–∞_, area under the curve extrapolated to infinity; AUMC_0–∞_, area under the first moment curve extrapolated to infinity; *C*_max_, maximum plasma concentration; *F*, bioavailability; *t*_1/2_ *z*, terminal half‐life; *T*_max_, time of *C*_max_; MRT_0–∞_, mean residence time extrapolated to infinity; *λ*z, terminal rate constant.

^a^ Harmonic mean and harmonic standard deviation.

**TABLE 3:** Absorption half‐life and rate constants of flunixin meglumine at the dosage of 3.3 mg/kg body weight following single intravenous (TD) administration in wool (dorset; *n* = 6) and hair (katahdin; *n* = 6) sheep using an open one‐ or two‐compartmental model.

Parameter	Unit	Wool (dorset) sheep				Hair (katahdin) sheep				*p*
		Mean	SD	Median	Range	Mean	SD	Median	Range	
*K*_01_	1/h	0.37	2.27	0.48	0.13–0.83	0.15	1.44	0.16	0.10–0.23	0.13
*t*_1/2abs_ ^a^	h	1.47	0.54	1.43	0.84–5.27	4.28	3.53	4.36	3.17–6.88	0.06
*K*_12_	1/h	0.023	1.68	0.02	0.01–0.05	0.025	1.43	0.02	0.02–0.04	0.33
*K*_21_	1/h	0.006	3.41	0.004	0.002–0.05	0.45	1.67	0.022	0.002–0.03	0.08
MAT	h	13.74	1.57	11.82	8.89–27.91	23.73	1.35	24.22	13.99–35.77	0.045

Abbreviations: *K*_01_, absorption rate constant; *K*_12_, transfer rate constants from compartment one to compartment two; *K*_21_, transfer rate constant from compartment two to compartment one; MAT, mean absorption time; *t*_1/2abs_, absorption half life.

^a^ Harmonic Mean and harmonic standard deviation.

## 4 Discussion

The present study was conducted to determine the pharmacokinetics of IV and TD flunixin in healthy adult female sheep of two different breeds. To the authors knowledge, there are no studies investigating the PK parameters of transdermal flunixin in sheep of variable wool types. Pharmacokinetic data have been reported for intramuscular (IM) and IV administration in sheep (Welsh et al. 1993). The labeled dose of transdermal flunixin formulation in cattle is 3.3 mg/kg. Based on previous studies performed in meat goats and cattle, a 3.3 mg/kg dose was used in the present study (Reppert, Kleinhenz, Montgomery, Bornheim, et al. 2019).

The pharmacokinetics of flunixin following IV administration in these sheep did not identify any significant differences between breeds. Mean concentrations at time 0 in wool sheep (25.9 μg/mL) and hair sheep (24.62 μg/mL) were similar to values reported in boer goats (21.89 μg/mL) and dairy bull calves (20.72 μg/mL) (Reppert, Kleinhenz, Montgomery, Bornheim, et al. 2019; Kleinhenz et al. 2018). The mean elimination half‐life (*t*_1/2_ *z*) following IV administration in wool (6.36 ± 1.13 h) and hair sheep (5.99 ± 1.54)was similar to values reported in cattle (5.44 h) and goats (6.032 h).

Bioavailability (*F*) is a measure of the systemic availability of a drug administered by a route other than IV and is determined by comparing the area under the plasma drug concentration curve versus time (AUC) for the extravascular formulation to the AUC for the IV formulation. AUC values for IV administration were 45.85 ± 22.29 h × μg/mL and 30.88 ± 3.77 h × μg/mL in wool versus hair sheep were not significantly different, but tended to be higher than previously reported in goats (32.79 h × μg/mL), alpacas (25.212 h × μg/mL) and 2–8 month old calves (14.96–9.00 h × μg/mL) (Reppert, Kleinhenz, Montgomery, Bornheim, et al. 2019; Kleinhenz et al. 2018; Reppert, Kleinhenz, Montgomery, Heiman, et al. 2019). Sources of this variability can be attributed to species/breed differences, sampling design as well as limit of detection of sample analysis. Ideally, the extrapolation portion of the AUC should remain as limited as possible. The % of AUC extrapolated for both wool and hair sheep was < 0.06% for IV and < 7.9% for TD. Wool sheep had significantly increased (*p* = 0.002) AUC_0–∞_ values (27.94 ± 1.22 h × μg/mL) than hair sheep (16.74 ± 1.18 h × μg/mL). Hair sheep were similar to AUC values reported in goats (12.18 h × μg/mL), higher than alpacas (6.315 h × μg/mL) and calves (8.52 h × μg/mL). There are two possible routes of drug penetration across the intact skin, through the stratum corneum or passage of molecules through sweat glands and across the hair follicles (Zaid Alkilani et al. 2015). Although there is limited comparative data on the stratum corneum thickness among goats, cattle, and sheep, this may influence differences noted in AUC. Goat skin showed less dermal papillae, less hair follicles, and less sebaceous glands than sheep, all of which may impact drug penetration and therefore absorption (Mohammed et al. 2022). The wool of sheep has a continuous coat of an emulsion consisting of sebum and sweat that can act as a solvent to improve or prohibit drug absorption from the skin (Pitman and Rostas 1981). Transdermal drug delivery is affected by physiological factors such as skin thickness, hydration, inflammation, pH, lipid content, densities of sweat glands and hair follicles, blood flow to skin, and physicochemical factors such as partition coefficient, molecular weight, and degree of ionization. Following IV administration, the coat type is irrelevant, but other breed‐related factors including hepatic/renal blood flow, metabolism, body composition/distribution, and other covariates may be responsible for PK differences. The current study design only allowed for evaluation of breed as a single covariate, and future studies are needed to evaluate other breed‐related factors.

Formulation of a transdermal product and its local effects should be considered as a potential source of variability across species. The product used in the current study contained several inert ingredients (menthol, glycerol, pyrrolidone, and glycol) that may have led to improved absorption in wool versus hair sheep, leading to decreased MAT (rate of drug absorption) in wool sheep compared to hair sheep in this study (Magnusson et al. 2001). Interindividual variability in bioavailability is likely attributed to the interindividual variability in MAT seen in this study (Weiss and Siegmund 2023). Identifying different covariates among breeds of sheep is important for understanding inter‐individual variability in drug absorption and therefore pharmacokinetics.

The mean bioavailability of TD flunixin was not different across breeds (48.76% ± 17.49% and 36.61% ± 4.33%) in wool and hair sheep, respectively, but was higher than noted in alpacas (25.05%) and meat goats (25.6%) (Reppert, Kleinhenz, Montgomery, Heiman, et al. 2019; Reppert, Kleinhenz, Montgomery, Bornheim, et al. 2019). Variability of the *F* in a population of animals is more clinically significant than the mean. Large variations in bioavailability may cause either subtherapeutic dosages or potentially overdosing, and small sample numbers and inclusion of only two breeds of sheep likely contributed to the wide range noted in the current study.

Increasing the sample size in this study could have enhanced the robustness of the analysis. This study was performed as a cross‐over study, but animals were not randomized for each treatment. Every enrolled sheep received both IV then TD flunixin. The lack of randomization is unlikely to affect the reported PK results but is a limitation of this study. Another factor to consider when applying the reported data in this study to clinical patients is the impact on environmental temperature. Previous studies were conducted in cattle in varying environmental temperatures that ranged from an average low of 15.3°F–20.1°F (the coldest study) to an average high of 80°F–100°F (the warmest study) but the current study was conducted inside a lab animal facilities housing unit at a controlled temperature (daily average 71°F). Absorption of flunixin administered topically to cattle was shown to vary as a function of environmental temperature. In the coldest temperatures, the time to reach peak plasma concentration (*T*_max_) increased to at least 4 h versus the average of 1.75 h in summer studies (Food and Drug Administration 2017). Further research is needed to determine environmental temperatures on flunixin absorption across different breeds (including wool and hair sheep).

In conclusion, the use of transdermal flunixin presents a significant advancement in pain management and anti‐inflammatory treatment for sheep. Its ease of administration may reduce stress and handling, improving both animal welfare and producer compliance. Increased plasma concentrations and prolonged elimination half‐life highlight its potential as a valuable therapeutic option for veterinarians and producers. Further research across different breeds and stages of production, including different ages of sheep before and after shearing, in addition to efficacy trials, is needed to further determine its usefulness in clinical patients.

## Author Contributions

D.A.M., J.L.H., R.E.B. contributed to study design. K.G.F., D.A.M., J.L.H. performed the experiments. D.A.M., J.L.H. performed data analysis. K.G.F. and D.A.M. composed the manuscript with help from R.E.B. and J.L.H. All authors have read and approved the final document.

## Conflicts of Interest

The authors declare no conflicts of interest.

## Supporting information


**Data S1:** jvp70015‐sup‐0001‐Tables.docx.

## Data Availability

The data that support the findings of this study are available from the corresponding author upon reasonable request.
