# Supplementary material for: Pharmacokinetics of Intravenous and Transdermal Flunixin Meglumine in Wool and Hair Sheep (Ovis aries)
Source: J Vet Pharmacol Ther. 2025 Aug 1;48(6):484–90. doi: 10.1111/jvp.70015 (PMC12598903; doi:10.1111/jvp.70015)
Supplement: Supplementary file 1 — Data S1: jvp70015‐sup‐0001‐Tables.docx. [file JVP-48-484-s001.docx]

Supplementary Tables

Supplementary Table 1. Intraday precision and accuracy of analytical method for flunixin sheep plasma samples

| Nominal concentration of flunixin (µg/mL) | Average observed concentration of flunixin (µg/mL) n=5 | Average recovery (%) | Coefficient of variation (%) |
| --- | --- | --- | --- |
| 0.001 | 0.0011 | 107.6 | 4.1 |
| 0.003 | 0.0027 | 90.1 | 6.0 |
| 0.03 | 0.031 | 103.2 | 5.0 |
| 0.1 | 0.11 | 107.8 | 5.4 |
| 0.3 | 0.3 | 100.0 | 4.8 |
| 0.7 | 0.67 | 96.1 | 4.8 |
| 3 | 2.93 | 97.7 | 4.6 |
| 7 | 7.04 | 100.6 | 7.6 |

Supplementary Table 2. Inter-day precision and accuracy of analytical method for flunixin sheep plasma samples

| Nominal concentration of flunixin (µg/mL) | Average observed concentration of flunixin (µg/mL) | Average recovery (%) | Coefficient of variation (%) |
| --- | --- | --- | --- |
| 0.0005 | 0.00046 | 91.08 | 11.9 |
| 0.001 | 0.00102 | 101.26 | 8.2 |
| 0.005 | 0.00512 | 102.38 | 5.76 |
| 0.01 | 0.01024 | 102.26 | 5.29 |
| 0.05 | 0.05142 | 102.88 | 5.74 |
| 0.1 | 0.10168 | 101.68 | 7.09 |
| 0.5 | 0.48862 | 97.74 | 3.70 |
| 1 | 1.008 | 100.8 | 2.52 |
|  |  |  |  |
| 0.05 | 0.0516 | 103.2 | 3.53 |
| 0.1 | 0.1015 | 101.47 | 5.29 |
| 1 | 0.9486 | 94.83 | 5.67 |
| 2 | 2.0419 | 102.07 | 4.58 |
| 5 | 4.4396 | 88.8 | 13.25 |
| 10 | 10.1755 | 101.73 | 3.23 |
